# A Systematic Review of the Efficacy of Bioactive Compounds in Cardiovascular Disease: Phenolic Compounds

**DOI:** 10.3390/nu7075177

**Published:** 2015-06-29

**Authors:** Oscar D. Rangel-Huerta, Belen Pastor-Villaescusa, Concepcion M. Aguilera, Angel Gil

**Affiliations:** Department of Biochemistry and Molecular Biology II, Centre of Biomedical Research, Institute of Nutrition and Food Technology “Jose Mataix”, University of Granada, Conocimiento Avenue, 18006 Granada, Spain; E-Mails: odrangel@ugr.es (O.D.R.H.); mbpastor.ugr@gmail.com (B.P.V.); caguiler@ugr.es (C.M.A.)

**Keywords:** bioactive food compounds, cardiovascular diseases, polyphenols, phenols, flavonols

## Abstract

The prevalence of cardiovascular diseases (CVD) is rising and is the prime cause of death in all developed countries. Bioactive compounds (BAC) can have a role in CVD prevention and treatment. The aim of this work was to examine the scientific evidence supporting phenolic BAC efficacy in CVD prevention and treatment by a systematic review. Databases utilized were Medline, LILACS and EMBASE, and all randomized controlled trials (RCTs) with prospective, parallel or crossover designs in humans in which the effects of BAC were compared with that of placebo/control were included. Vascular homeostasis, blood pressure, endothelial function, oxidative stress and inflammatory biomarkers were considered as primary outcomes. Cohort, ecological or case-control studies were not included. We selected 72 articles and verified their quality based on the Scottish Intercollegiate Guidelines Network, establishing diverse quality levels of scientific evidence according to two features: the design and bias risk of a study. Moreover, a grade of recommendation was included, depending on evidence strength of antecedents. Evidence shows that certain polyphenols, such as flavonols can be helpful in decreasing CVD risk factors. However, further rigorous evidence is necessary to support the BAC effect on CVD prevention and treatment.

## 1. Introduction

The prevalence of cardiovascular disease (CVD) is rising and is the prime cause of death in all developed countries [1], and one of the most important health issues in developing countries [2]. While some risk factors cannot be changed, such as family history, ethnicity and age, detection and control of modifiable factors such as, blood pressure (BP), high cholesterol, obesity, type 2 diabetes (T2D) or unhealthy diets can help to prevent intermediate risk CVD processes like inflammation or oxidative stress. Thus, primary prevention of CVD by identifying and treating at-risk individuals remains a major public-health priority. A healthy life style is the main pre-emptive approach [3,4].

Dietary habits are quite different around world; nevertheless, certain consumption patterns are common worldwide, inclusion of fruits and vegetables or products like cocoa, coffee or condiments is a merging point. Bioactive compounds (BAC) are “extra nutritional” constituents that are present in small quantities in plant products and lipid rich foods [5]. The growing body of scientific evidence indicates that certain BAC play a beneficial role in CVD prevention [6,7,8,9,10,11]. BAC oral supplements along with a usual diet can increase the intake of ingredients reputed to have clinical benefits. These supplements are, usually, an addition to the healthy diet, and not as a conventional food or the sole item of a meal [12].

Putative beneficial biological effects such as antilipidemic, antihypertensive, anti-glycaemic, antithrombotic and anti-atherogenic effects are attributed to BAC. In the present study, the main goal was to examine the scientific evidence of BAC in the prevention and treatment of CVD by a systematic review of randomized clinical trials (RCTs). The BAC considered in this review were all those related to the phenolic compounds.

Phenolic compounds such as stilbenes like the resveratrol (3,5,4′-trihydroxystilbene) can be found principally in the skin of grapes and are produced in other plants, such as peanuts [6]. Red wine is a rich source of resveratrol and is thought to confer the cardio protective effects associated with moderate consumption of wine [13]. Within the catechols family, curcuminoids are multifunctional natural compounds found in native Indonesian plants, with promising cardio protective and anti-inflammatory properties and mainly present in the dried rhizomes of *Curcuma longa L*. (commonly known as turmeric) [14].

In relation to polyphenols, there are six basic subclasses of flavonoids: flavones, anthocyanins, flavanones, flavonols, isoflavones, and the flavanols, including the flavanol oligomers, the proanthocyanidins that are further subdivided into 16 species including the procyanidins, oligomers of the flavan-3-ols catechin and epicatechin, and the prodelphinidins, oligomers of the gallocatechins [15]. In this review, we utilized equations to divide the results according to the most relevant classes.

Specifically, we examined the effects of BAC on BP, lipid profile [triacylglycerol (TAG), cholesterol, high and low density lipoproteins(HDL and LDL)], carbohydrate (CHO) metabolism (glucose, insulin, and insulin resistance (IR)), oxidative stress, inflammation and endothelial function (EF). Furthermore, we gave a recommendation for consumption based on the evidence grade according to Scottish Intercollegiate Guidelines Network (SIGN) [16].

## 2. Methodology

We developed a literature search in Medline by PubMed (U.S. National Library of Medicine and the NIH), and in LILACS and EMBASE, including publications in English, Spanish and Portuguese until December 2014. Studies eligible for this review included: randomized controlled trials (RCTs) in healthy and unhealthy adults, with prospective, parallel or crossover designs, with full text, and whose primary outcomes were vascular homeostasis, BP, oxidative stress and/or inflammatory biomarkers; we excluded those studies with cohort, ecological or case-control design, those which analysed a drug, or when BAC were combined with other compounds. However, there was no restriction on publication type or sample size.

### 2.1. Search Equation

**Figure 1:**
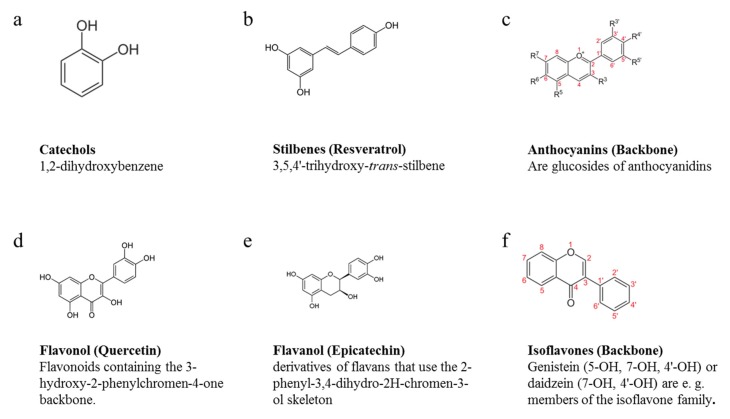
Chemical diversity polyphenols. Simple phenols are represented by (**a**) catechols and (**b**) stilbenes, and polyphenols in (**c**) anthocyanins, (**d**) flavonols, (**e**) flavanols and (**f**) isoflavones.

Due to the diversity of the chemical structures of phenol compounds (Figure 1), the type of BAC can present different effects in CVD; consequently, we evaluated the more relevant groups. We included different keywords in the search equation of bioactive compounds, including: Phenols (*stilbenes*, *catechols*, *flavonoids*, *anthocyanins*, *flavanones*, *isoflavones*, *polyphenols*, *phenolic acids*, *gallic acid and hydroquinones*). We combined the MeSH term “cardiovascular diseases” with each bioactive compound as MeSH Major Topic, together with NOT “review” (Publication Type) in PubMed. However, equations in the Spanish language were used when the search was carried out in LILACS *i.e.*, (tw:(polifenoles)) AND (tw:(enfermedad cardiovascular)) AND NOT (tw:(revisión)). When consulting the database EMBASE, the equations were elaborated as “catechols”/mj AND “cardiovascular diseases”/mj “NOT review”. Articles published before 1990 were discarded because they did not comply the inclusion criteria stablished.

After the review process by proofreading staff, we included four additional articles. Three of them were not located by our search criteria; the other appeared in our initial search, but its main outcome in relation to exercise did not comply with the requirement for inclusion in the review. Nevertheless, after a second approach by the proofreading staff, we also decided to include it (see footnotes in the Table 1, Table 2 and Table 3).

### 2.2. Selection and Evaluation

First, both titles and abstracts were identified independently by two reviewers, for exclusion of those articles that did not fit with the language, date, subject matter, design and outcomes established. Then, full-text publications were classified by pathologies according to outcomes analysed in each study.

Moreover, RCTs were finally selected if they obtained a score between 3 and 5 according to the Jadadscale [17]. This method attempts to reduce bias for RCTs, ensuring a certain quality in the evidence; it took into account if they were randomized, blinded and provided detailed information about patients.

Furthermore, we verified the quality of selected articles by the Scottish Intercollegiate Guidelines Network (SIGN) [16]. Diverse quality levels of scientific evidence are established according to two features: the design and bias risk of a study. The levels are from 1++, when the information is considered as high quality, to 4 when the information is considered as very low quality. Signs are used to reporting with reference to compliance degree of key criteria associated with potential bias (1++, 1+, 1-, 2++, 2+, 2-, 3, and 4). Additionally, we included a grade of recommendation, based on the evidence strength of the antecedents, whose levels are A, B, C, D, with “A” being highly recommended and “D” not recommended. These grades of recommendation by SIGN guidelines are equivalent to those designated by the Food and Agriculture Organization of the United Nations/World Health Organization (FAO/WHO), as evidence criteria: convincing, probable, possible and insufficient [18].

## 3. Results and Discussion

**Figure 2:**
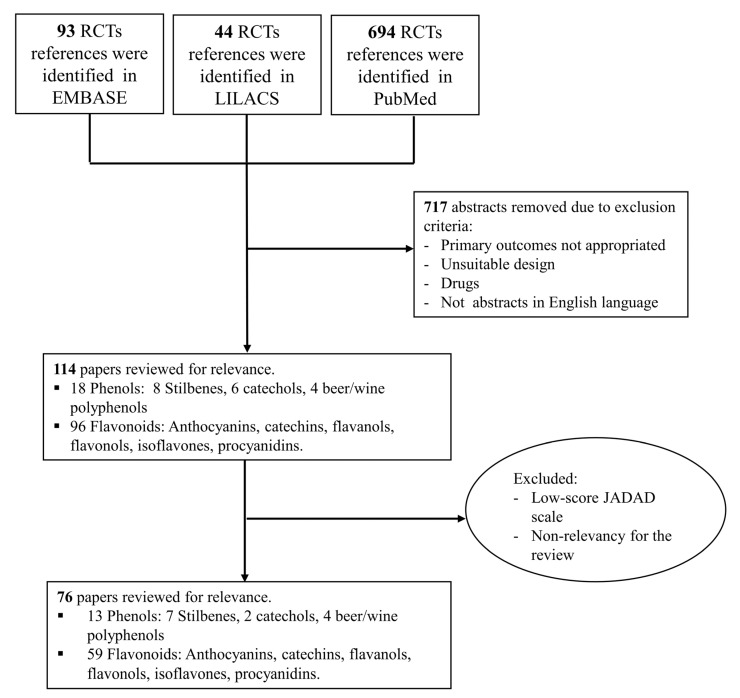
Review Flow Diagram.

In total, 831 RCT’s were found using the equations proposed in the different databases (EMBASE, LILACS and PubMed). We excluded 717 due to obvious irrelevance, leaving 114 papers in full to read (Figure 2). After papers were read and evaluated using the Jadad scale, 76 articles were selected for the final review, and are included in Table 1, Table 2 and Table 3.

**Table 1:** RCTs of phenolic compounds (catechols, stilbenes and beer/wine) in CVD risk.

Group (Class)	Author/Date	Jadad Score	Design (Follow up)	(n) Population	Intervention	Outcomes	Significant Results
Phenols (Stilbenes)	Wong *et al.* (2011) [19]	5	2B, X (1 h)	(19) Overweight/obese + ↑ BP men or post-menopausal women	30 mg, 90 mg, 270 mg RSV *vs.* PCB	EF	↑ EF, more with highest dose
Phenols (Stilbenes)	Wong *et al.* (2013) [20] *	5	2B, X (1 h)	(28) obese subjects	Acute intervention: 75 mg/trans-resveratrol (Resvida) *vs.* PCB after chronic intervention	FMD	↑ FMD
Phenols (Stilbenes)	Wong *et al.* (2013) [20] *	5	2B, X (6 weeks)	(28) obese subjects	75 mg/day trans-resveratrol (Resvida) *vs.* PCB	BP, AR, BMI, FMD	↑ FMD
Phenols (Stilbenes)	Bo *et al.* (2013) [21]	5	2B, X (60 days(wash-out 30 days))	(50) Healthy smokers	500 mg RSV/d *vs.* PCB	BP, Anthropometry, lipids profile, CHO metabolism, TAS, hsCRP,	↓ hsCRP, TAG, ↑ TAS
Phenols (Stilbenes)	Militaru *et al.* (2013) [22]	3	2B, Ctrl, PA (60 days)	(166) BMI 24–27 kg/m^2^, stable angina pectoris	20 mg/day RSV, 20 mg/day RSV + 112 mg/day CF, 112 mg/day CF	Lipids profile, hsCRP, left ventricular function markers	↓ TC, TAG greater in RSV, hsCRP greater in CF, NT-proBNP more effective RSV+CF
Phenols (Stilbenes)	Tomé-Carneiro *et al.* (2013) [23]	4	3B, PCB (1 year)	(75) Stable CAD patients	350 mg/day GE, 350 mg/day GE-RES *vs.* PCB (6 months); double dose next 6 months	PBMCs, inflammatory and fibrinolytic biomarkers	↑ adiponectin, ↓ PAI-1, significantly activated or inhibited 6 key inflammation-related transcription factors in PBMCs
Phenols (Stilbenes)	Tomé-Carneiro *et al.* (2012) [24]	4	3B, PCB (6 months)	(75) Primary prevention of CVD	350 mg/day GE, 350 mg/day GE-RES *vs.* PCB	Lipids profile, oxidized LDL	↓ LDLc, ApoB, LDLox and LDLox/ApoB ratio, ↑ nonHDLc/ApoB ratio in GE-RES
Phenols (Stilbenes)	Tomé-Carneiro *et al.* (2013) [25]	5	3B, PCB, dose–response (1 year)	(35) T2D, HT with CAD	350 mg/day GE, 350 mg/day GE-RES *vs.* PCB (6 months); double dose next 6 months	PBMCs, inflammatory, fibrinolytic biomarkers	↓ CCL3, IL-1β, TNF-α expression, ↑ transcriptional repressor LRRFIP-1 in PBMCs with GE-RES
Phenols (Stilbenes)	Tomé-Carneiro *et al.* (2012) [26]	4	3B, PCB (1 year)	(75) Primary prevention of CVD	350 mg/day GE, 350 mg/day GE-RES *vs.* PCB (6 months); double dose next 6 months	Inflammatory and fibrinolytic biomarkers	↓ CRP, TNF-α, PAI-1, IL-6/IL-10 ratio, sICAM ↑ IL-10, adiponectin in GE-RES
Phenols (Catechols)	Alwi *et al.* (2008) [27]	4	2B, PCB (2 months)	(75) ACS patients	45 mg/day, 90 mg/day or 180 mg/day curcumin *vs.* PCB	Lipids profile	Not significant effect
Phenols (Catechols)	Chuengsamarn *et al.* (2014) [28]	5	2B, PCB (6 months)	(240) T2D patients	750 mg/day curcumin *vs.* PCB	BP, anthropometry, lipids profile, adiponectin, leptin, CHO metabolism, PWV, uric acid	↓ PWV, HOMA, TAG, uric acid, abdominal obesity and leptin, ↑ adiponectin.
Polyphenols (Wine/beer)	Botden *et al.* (2012) [29]	4	2B, PCB, three-period X (4 weeks)	(61) HT subjects	280 mg/day red wine polyphenols or 560 mg/day red wine polyphenols *vs.* PCB	BP	No significant effect
Polyphenols (Wine/beer)	Chiva-Blanch *et al.* (2014) [30]	4	2B, PCB, X (4 weeks)	(36) High risk of CVD males	Beer (30 g alcohol/day), the equivalent amount of polyphenols in the form of non-alcoholic beer, or gin (30 g alcohol/day)	Circulating endothelial progenitor cells and EPC-mobilizing factors	Beer and non-alcoholic beer interventions, ↑-circulating EPC. No significant differences were observed after the gin period
Polyphenols (Wine/beer)	Chiva-Blanch *et al.* (2012) [31]	3	X (4 weeks)	(67) High risk of CVD males	Red wine (30 g alcohol/day), the equivalent amount of dealcoholized red wine, or gin (30 g alcohol/day)	BP and plasma nitric oxide	Dealcoholized red wine ↓ DBP and SBP
Polyphenols (Wine/beer)	Chiva-Blanch *et al.* (2012) [32]	3	X (4 weeks)	(67) High risk of CVD males	Red wine (30 g alcohol/day), the equivalent amount of dealcoholized red wine, or gin (30 g alcohol/day)	Inflammatory biomarkers	Alcohol ↑ IL-10 and ↓ macrophage-derived chemokine concentrations. Phenolic compounds of Red wine ↓ serum concentrations of ICAM-1, E-selectin, and IL-6

2B, double-blinded, 3B, triple-blinded, ACS, acute coronary syndrome; Apo, apolipoprotein; BMI, body mass index; BP, blood pressure; CAD, chronic artery disease; CHO, carbohydrate; CCL-3, chemokine (C–C motif) ligand 3 CRP, C-reactive protein; hsCRP, high sensitivity c-reactive protein; Ctrl, control, CVD, cardiovascular disease; EF, endothelial function; EPC, endothelial progenitor cells; FMD, flow mediated dilation; GE, grape extract; GE-RES, grape extract containing RSV (8mg); HDLc, high-density lipoprotein cholesterol; HOMA, homeostasis model assessment; HT, hypertension; sICAM, soluble intercellular adhesion molecule; IL, interleukin; LDLc, low-density lipoprotein cholesterol; LDLox, oxidized LDL; LRRFIP-1, leucine rich repeat (in FLII) interacting protein 1; NT-proBNP, N-terminal prohormone of brain natriuretic peptide; PAI-1, plasminogen activator inhibitor-1; PCB, placebo, PBMCs, peripheral blood mononuclear cells; PWV, pulse wave velocity; RSV, resveratrol; TAG, triacylglycerols; TC, total cholesterol; TNF-α,tumour necrosis factor alpha; T2D, type 2 diabetes; X, crossover design. * Included after proofreading.

**Table 2:** RCTs of polyphenols (anthocyanins, catechins, flavanols and flavonols) in CVD risk.

Group (Class)	Author/ Date	Jadad Score	Design (Follow up)	(n) Population	Intervention	Outcomes	Significant Results
Flavonoids (Anthocyanins)	Kuntz (2014) [33]	4	2B, PCB, X (14 days)	(30) Healthy females	330 mL/day beverages (PCB, juice or smoothie with 8.9, 983.7 and 840.9 mg/L ACN, respectively)	Inflammatory and oxidative stress biomarkers	↑ SOD and CAT after ACN. ↓ MDA after ACN ingestion.
Flavonoids (Anthocyanins)	Curtis *et al.* (2009) [34]	5	PCB, PA (12 weeks)	(57) Postmenopausal women	500 mg/day ACN *vs.* PCB	BP, CHO metabolism, lipids profile, inflammatory biomarkers, platelet reactivity	No significant effect
Flavonoids (Anthocyanins)	Hassellund *et al.* (2013) [35]	5	2B, PCB, X (4 weeks)	(31) Pre-hypertensive males	640 mg/day ACN *vs.* PCB	Lipids profile, CHO metabolism, inflammatory and oxidative stress biomarkers	↑ HDLc and glucose after anthocyanin *versus* PCB treatment. No effects were observed on inflammation or oxidative stress *in vivo*, except for vWf
Flavonoids (Anthocyanins)	Dohadwala *et al.* (2011) [36]	4	Open-label, (2 and 4 hour acute study)	(15) CAD subjects	835 mg total polyphenols, 94 mg anthocyanins *vs.* PCB	Vascular function	No significant effect
Flavonoids (Anthocyanins)	Dohadwala *et al.* (2011) [36]	4	X, 2B, PCB (4 weeks, 2 week washout)	(44) CAD subjects	835 mg total polyphenols, 94 mg anthocyanins *vs.* PCB	Vascular function	↓ Carotid femoral pulse wave activity
Flavonoids (Catechins)	Miyazaki *et al.* (2013) [37]	4	2B, PCB (14 weeks)	(52) Healthy subjects	630.9 mg/day Green Tea Catechins *vs.* Ctrl	CVD risk markers	No significant effect
Flavonoids (Catechins)	de Maat *et al.* (2000) [38]	3	1B, PCB, PA (4 weeks)	(64) Healthy subjects	Black tea (3 g/day), green tea (3 g/day), green tea polyphenol isolate capsules (3.6 mg/day) and mineral water.	Inflammatory and endothelial markers	Negative correlation between the levels of the antioxidant β-carotene and the inflammation markers IL6 and fibrinogen
Flavonoids (Catechins)	Widmer *et al.* (2013) [39]	3	2B, Ctrl (4 months)	(52) Early atherosclerosis	30 mL/day simple Olive Oil *vs.* 30 mL/day of EGCG-supplemented Olive Oil	EF, inflammation and oxidative stress	Only significant when merging data of both groups the EF was improved.
Flavonoids (Catechins)	Nagao *et al.* (2007) [40]	4	2B, PA (12 weeks)	(240) Visceral fat-type obesity	Green tea containing 583 mg/day catechins (catechin group) *vs.* 96 mg/day catechins (Ctrl group)	Anthropometric measurements, body fat composition and CVD risk	↓ body weight, BMI, body fat ratio, body fat mass, waist circumference, hip circumference, visceral fat area, and subcutaneous fat area, SBP, LDLc
Flavonoids (Flavanols)	Farouque *et al.* (2006) [41]	5	2B, PCB (6 weeks)	(40) Healthy males	Flavanol-rich chocolate bar and cocoa beverage (total flavanols, 444 mg/day) *vs.* matching isocaloric PCBs (total flavanols, 19.6 mg/day)	EF and adhesion molecules	No significant effect
Flavonoids (Flavanols)	Berry *et al.* (2010) [42] *	4	2B, X (2 h, 3–7 days washout)	(21) overweight/obese subjects	HF, 701 mg or LF, 22 mg cocoa	BP, HR, FMD	↑ DBP after exercise were attenuate by HF, improvement of FMD with HF
Flavonoids (Flavanols)	Davison *et al.* 2008 [43] *	4	2B, PCB, PA (12 weeks)	(98) overweight/obese subjects	902 mg cocoa flavanols/day *vs.* 36 mg cocoa flavanols/day With/without exercise protocol	BP, HDLc, LDLc, TG, HOMA, FMD	↑ FMD at 6 and 12 weeks with HF *vs.* LF, ↑ DBP, BP mean, improvement in HOMA (independent of exercise)
Flavonoids (Flavanols)	West *et al.* (2014) [44]	3	2B, PCB, X (4 weeks, 2 weeks washout)	(30) Middle-aged overweight	37 g/day of dark chocolate and a sugar-free cocoa beverage (total flavanols = 814 mg/day) *vs.* low-flavonol chocolate and cocoa free beverage (total flavanols = 3 mg/day)	EF, BP	↑ Basal and peak diameter of the brachial artery and basal blood flow volume.
Flavonoids (Flavanols)	Faridi *et al.* (2008) [45]	4	X, Ctrl, 1B (1 days, 7 days washout)	(45) Overweight subjects	Solid dark chocolate bar (821 mg flavanols) *vs.* cocoa-free PCB bar (0 mg flavanols)	EF, BP	Solid dark chocolate improved EF; also ↓ BP
Flavonoids (Flavanols)	Faridi *et al.* (2008) [45]	4	X, Ctrl, 1B (1 days, 7 days washout)	(44) Overweight subjects	Sugar-free cocoa (805.2 mg flavanols), sugared cocoa (805.2 mg flavanols), *vs.* PCB (0 mg flavanols).	EF, BP	Liquid cocoa ingestion improved EF; sugar-free cocoa ↓ BP
Flavonoids (Flavanols)	Davison *et al.* (2010) [46]	3	2B, PA (6 weeks)	(52) Men and postmenopausal women with untreated mild HT	33, 372, 712 or 1052 mg/day of cocoa flavanols	24-h BP	No significant effect
Flavonoids (Flavanols)	Grassi *et al.* (2008) [47]	3	X, Ctrl, 1B (15 days)	(19) HT with Impaired glucose tolerance	Flavonol-rich dark chocolate (110.9 mg epicatechin, 36.12 mg catechin, 2.5 mg quercetin, 0.03 mg kaempferol, and 0.2 mg isorhamnetin)/d or flavonol-free white chocolate (0.04 mg/day catechins)	EF, IR, β-cell function, BP, CRP, TC	↓ IR, BP, TC, LDLc. ↑ insulin sensitivity, EF
Flavonoids (Flavanols)	Flammer *et al.* (2012) [48]	3	2B, PCB (2 hours)	(20) CHF patients	40 g Flavonol rich chocolate (624 mg total flavanols) *vs.* 28.4 g Ctrl chocolate (0 mg flavanols	EF and platelet function in the short term	Improvement of vascular function in patients with CHF
Flavonoids (Flavanols)	Flammer *et al.* (2012) [48]	3	2B, PCB (2 and 4 weeks)	(20) CHF patients	40 g/day Flavonol rich chocolate (624 mg total flavanols) *vs.* 28.4 g/day Ctrl chocolate (0 mg flavanols)	EF and platelet function in long term by FMD	Improvement of vascular function in patients with CHF
Flavonoids (Flavanols)	Heiss *et al.* (2010) [49]	3	Ctrl, 2B, X (30 days)	(16) CAD patients	High-flavanol intervention (375 mg/day) and a macronutrient- and micronutrient-matched low-flavanol intervention (9 mg/day) twice daily	EF and enhancement and function of circulating angiogenic cells	↑ EF, CD34+/KDR+-Circulating angiogenic cells. ↓ SBP
Flavonoids (Flavanols)	Horn *et al.* (2013) [50]	3	2B, X (30 days)	(16) CAD patients	High-flavanol intervention (375 mg/day) and a macronutrient- and micronutrient-matched low-flavanol intervention (9 mg/day) twice daily	Circulating endothelial micro particles, markers of endothelial integrity, EF	↑ Endothelial micro-particles and EF. Improvement of endothelial integrity
Flavonoids (Flavanols)	Balzer *et al.* (2008) [51]	5	2B, PCB, three-period X (2 h)	(10) Diabetic subjects	Single-dose ingestion of cocoa, containing increasing concentrations of flavanols (75, 371, and 963 mg)	EF	Single ingestion of flavanol-containing cocoa was dose-dependently acute increases in circulating flavanols and EF
Flavonoids (Flavanols)	Balzer *et al.* (2008) [51]	5	2B, PCB, PA (30 days)	(41) Diabetic subjects	963 mg/day Flavanol-rich cocoa *vs.* nutrient-matched Ctrl (75 mg/day flavanols)	EF	Flavanol-containing cocoa ↑ baseline EF
Flavonoids (Flavonols)	Larson *et al.* (2012) [52]	3	2B, PCB, X (1 days, 7 days washout)	(5) Healthy males	1095 mg quercetin aglycone *vs.* PCB	Angiotensin-converting enzyme, endothelin-1, BP	No significant effect
Flavonoids (Flavonols)	Conquer *et al.* (1998) [53]	3	2B (28 days)	(27) Healthy subjects	4 capsules (1.0 g quercetin/day) *vs.* rice flour PCB	BP, lipids profile, thrombogenic risk factors	No significant effect
Flavonoids (Flavonols)	Suomela *et al.* (2006) [54]	3	2B, PCB, X (4 weeks, 4 weeks washout)	(14) Healthy males	Oat meal with 78 mg/day flavonol aglycones (sea buckthorn) *vs.* Ctrl	CVD risk markers	No significant effect
Flavonoids (Flavonols)	Edwards *et al.* (2007) [55]	3	2B, PCB, X (28 days)	(41) Prehypertension and hypertension	730 mg quercetin/day *vs.* PCB	BP, oxidative stress	↓ BP in hypertensive group
Flavonoids (Flavonols)	Larson *et al.* (2012) [52]	3	2B, PCB, X (1 days, 2 days washout)	(12) HT stage 1 males	1095 mg quercetin aglycone *vs.* PCB	Angiotensin-converting enzyme, endothelin-1, BP	↓ BP in Hypertensive men

8-iso-PGF2α, 8-iso-prostaglandin F2α; 1B, one-blind, 2B, double-blinded; ACN, anthocyanins; Apo, apolipoprotein; BMI, body mass index; BP, blood pressure; CAD, chronic artery disease; CAT, catalase; CHF, chronic heart failure; CHO, carbohydrate; CRP, C-reactive protein; hsCRP, high sensitivity c-reactive protein; Ctrl, control, CVD, cardiovascular disease; DXA, Dual-energy X-ray absorptiometry; EF, endothelial function; EGCG, epigallocatechin gallate; ESRD, European and North American end-stage renal disease; FM, fat mass; FFM, fat-free mass; FMD, flow mediated dilation; HDLc, high-density lipoprotein cholesterol; HOMA, homeostasis model assessment; HR, heart rate; HT, hypertension; sICAM, soluble intercellular adhesion molecule; IGF-1, insulin-like growth factor-1; IR, insulin resistance; IL, interleukin; LDLc, low-density lipoprotein cholesterol; MDA, malonaldehyde; MPFF, micronized purified flavonoid fraction; MPI, milk protein isolate; NTG, nitro-glycerine-mediated dilation; PA, parallel design, PAI-1, plasminogen activator inhibitor-1; PCB, placebo, PBMCs, peripheral blood mononuclear cells; PS, plant sterols; PWV, pulse wave velocity; QUICKI, quantitative insulin sensitivity check index; SBP, systolic blood pressure; SOD, superoxide dismutase; TC, total cholesterol; TGF, transforming growth factor; T2D, type 2 diabetes; VCAM, soluble vascular cellular adhesion molecule; vWf, von Willebrand factor; X, crossover design. * Included after proofreading.

**Table 3:** RCTs of polyphenols (isoflavones and procyanidins) in CVD risk.

Group (Class)	Author/Date	Jadad Score	Design (Follow up)	(n) Population	Intervention	Outcomes	Significant Results
Flavonoids (Isoflavones)	McVeigh *et al.* (2006) [56]	3	1B, X (57 days, 4 weeks washout)	(35) Healthy males	Milk protein isolate (MPI), low-isoflavone soy protein isolate (low-iso SPI; 1.64 ± 0.19 mg aglycone isoflavones/day), and high-isoflavone SPI (high-iso SPI; 61.7 ± 7.4 mg aglycone isoflavones/day)	Lipids profile	↓ TC/HDLc, LDLc/HDLc, and Apo B/Apo A-I with both SPI treatments than with MPI treatment
Flavonoids (Isoflavones)	Sanders *et al.* (2002) [57]	3	X (17 days, 25 days washout)	(22) Healthy subjects	56 *vs.* 2 mg isoflavones/day	Lipids profile, fibrinogen, and active TGF-β, factor VII coagulant and PAI-1	↑ HDL and Apo A1 in high-isoflavone
Flavonoids (Isoflavones)	Thorp *et al.* (2008) [58] *	5	2B, PCB, X (6 weeks)	(91) Hypercholesterolemia	24 g SP+70–80 mg ISOs (diet S) *vs.* 12 g SP + 12 g dairy protein (DP) + 70–80 mg ISOs (diet SD) *vs.* 24 g DP without ISOs (diet D)	HDLc, LDLc, TC	No significant effect
Flavonoids (Isoflavones)	Atkinson *et al.* (2004) [59]	5	2B, PCB (12 months)	(205) Female	43.5 mg red clover-derived isoflavones/day *vs.* PCB	Lipids profile, BP, fibrinogen and PAI-1	No significant effect
Flavonoids (Isoflavones)	Marini *et al.* (2010) [60]	5	2B, PCB (24 months)	(138) Females with low bone mass	54 mg/day genistein aglycone *vs.* PCB	Lipids profile, CHO metabolism, HOMA, fibrinogen, osteoprotegerin and homocysteine	↓ fasting glucose and insulin, HOMA, fibrinogen and homocysteine
Flavonoids (Isoflavones)	Hodis *et al.* (2011) [61]	5	2B, PCB (2 years)	(350) Postmenopausal women	25 g/day soy protein (91 mg/day aglycone isoflavone equivalents) *vs.* PCB	Atherosclerosis progression	No significant effect
Flavonoids (Isoflavones)	Atteritano *et al.* (2007) [62]	5	2B, PCB (24 months)	(191) Postmenopausal women	54 mg/day genistein *vs.* PCB	Lipids profile, CHO metabolism, HOMA, fibrinogen, sVCAM-1, sICAM-1, 8-iso-PGF2α, and osteoprotegerin	↓ Fasting glucose and insulin as well as HOMA, fibrinogen, 8-iso-PGF2α, sICAM-1, and sVCAM-1. ↑ Serum osteoprotegerin
Flavonoids (Isoflavones)	Garrido *et al.* (2006) [63]	3	PCB (12 weeks)	(29) Postmenopausal women	100 mg/day isoflavones *vs.* PCB	Lipids profile, CHO metabolism and platelet thromboxane A2 receptor density. BP, BMI, subcutaneous fat	↓ Thromboxane A2 after the experimental treatment.
Flavonoids (Isoflavones)	Hall *et al.* (2005) [64]	4	2B, PCB, X (8 weeks, 8 weeks washout)	(117) Postmenopausal women	Isoflavone-enriched (genistein-to-daidzein ratio of 2:1; 50 mg/day) *vs.* PCB cereal	Inflammatory and vascular homeostasis biomarkers	↓ CRP
Flavonoids (Isoflavones)	Rios *et al.* (2008) [65]	3	2B, PCB (6 months)	(47) Postmenopausal women	40 mg/day isoflavone *vs.* casein PCB	Lipids profile	No significant effect
Flavonoids (Isoflavones)	Villa *et al.* (2009) [66]	3	PCB (24 weeks)	(50) Postmenopausal women	54 mg/day genistein *vs.* PCB	Anthropometric measures, lipid profile, CHO metabolism and C-peptide evaluation, IR and EF	HOMA and fasting glucose levels significantly improved
Flavonoids (Isoflavones)	Liu *et al.* (2012) [67]	4	2B, PCB (6 months)	(180) Postmenopausal women	15 g/day soy protein and 100 mg/day isoflavone (Soy group), *vs.* 15 g/day milk protein and 100 mg/day isoflavone (Iso group) *vs.* 15 g/day milk protein (PCB)	Lipids profile, inflammatory markers and composite cardiovascular	No significant effect
Flavonoids (Isoflavones)	Yang *et al.* (2012) [68]	3	Open-labelled, prospective (24 week)	(130) Healthy Taiwanese postmenopausal women	35 mg/day *vs.* 70 mg/day soy extractª	Lipids profile	↓ TC, LDLc in patients with TC >200 mg/dL
Flavonoids (Isoflavones)	Liu *et al.* (2013) [67]	5	2B, PCB (6 months)	(270) Pre-hypertensive women	40 g/day soy flour (whole soy group), 40 g/day low-fat milk powder + 63 mg/day daidzein (daidzein group), *vs.* 40 g/day low-fat milk powder (PCB)	Anthropometric indicators and body composition	No significant effect
Flavonoids (Isoflavones)	Aubertin-Leheudre *et al.* (2008) [69]	3	2B, PCB (6 months)	(50) Obese postmenopausal women	70 mg/day isoflavones *vs.* PCB	Body composition (DXA), and Lipid profile and CHO metabolism	No significant effect
Flavonoids (Isoflavones)	Choquette *et al.* (2011) [70]	4	2B, PCB (6 months)	(100) Overweight to obese postmenopausal women	PCB or isoflavones (70 mg/day) or exercise + PCB or exercise + isoflavones (70 mg/day). Exercise consisted of three weekly sessions of resistance training and aerobics	Body composition, lipids profile, CHO metabolism and HOMA.	No significant effect
Flavonoids (Isoflavones)	Aubertin-Leheudre M *et al.* (2007) [71]	3	2B, PCB (12 months)	(56) Obese postmenopausal women	70 mg/day isoflavones^b^ (+weight loss exercise program from the 6 months) *vs.* PCB	Anthropometry, lipids profile, CHO metabolism, CRP	↓ body weight, BMI, total and abdominal FM (kg and %), ↑ FFM/FM ratio with exercise program
Flavonoids (Isoflavones)	Hodgson *et al.* (1999) [72]	3	2B, PCB, PA (8 weeks)	(59) High-normal BP	55 mg/day isoflavonoid *vs.* PCB	8-iso-PGF2α	No significant effect
Flavonoids (Isoflavones)	Sagara *et al.* (2004) [73]	3	PCB, 2B, PA (5 weeks)	(61) Men with relatively higher BP or TC	Diets containing at least 20 g/day soy protein + 80 mg/day isoflavones *vs.* PCB diets	BP and Lipid profile	↓ BP, TC and non-HDLc and ↑ HDLc.
Flavonoids (Isoflavones)	Clerici *et al.* (2007) [74]	4	Ctrl, PA (8 weeks)	(62) Hypercholesterolemia	80 g serving/d (33 mg/day isoflavones + negligible soy protein + led to a serum isoflavone concentration of 222 +/- 21 nmol/L) *vs.* Ctrl group	Lipids profile, hsCRP, urinary 8-iso-PGF2α, and EF	↓ LDLc, TC
Flavonoids (Isoflavones)	Meyer *et al.* (2004) [75]	3	PCB, X (5 weeks, without washout)	(23) Mildly hypercholesterolemic and/or hypertensive	Soy-based milk (30 g/day soy protein + 80 mg/day isoflavones) + yoghurt (treatment) *vs.* equivalent dairy products (Ctrl)	BP, arterial compliance, lipid profile, fatty acids	No significant effect
Flavonoids (Isoflavones)	Jenkins *et al.* (2002) [76]	3	1B (1 month, 2 weeks washout)	(41) Postmenopausal women with hypercholesterolemia	A low-fat dairy food Ctrl diet, high- (50 g soy protein and 73 mg isoflavones/day), low- (52 g soy protein and 10 mg isoflavones/day) isoflavone soy food diets	BP, lipids profile, oxidized LDL, calculated CAD risk	Soy diets ↓ TC estimated CAD risk, TC/HDLc, LDLc/HDLc, ApoB/A-I. Blood lipid and BP changes, the calculated CAD risk ↓ with the soy diets
Flavonoids (Isoflavones)	Blum *et al.* (2003) [77]	4	2B, PCB, X (6 weeks, 1 month washout)	(24) Postmenopausal women with hypercholesterolemia	25 g/day soy protein *vs.* PCB	Vascular inflammation biomarkers	No significant effect
Flavonoids (Isoflavones)	Teede *et al.* (2006) [78]	3	Ctrl, X (3 months)	(41) Hypertensive postmenopausal	Soy cereal (40 g/day soy protein + 118 mg/day isoflavones) *vs.* gluten PCB cereal	BP, arterial function	↑ 24 hour HR, area under curve of 24 h SBP
Flavonoids (Isoflavones)	Cicero *et al.* (2013) [79]	4	Ctrl, 1B prospective study with PA (12 weeks)	(40) Mildly dyslipidemic postmenopausal women	60 mg/day soy isoflavones + 500 mg/day berberine *vs.* PCB (1 tablet/d)	BP, HOMA, lipids profile, metalloproteinase	Isoflavones-berberine experienced a significant improvement in plasma lipid and metalloproteinase serum levels.
Flavonoids (Isoflavones)	Curtis *et al.* (2013) [80]	5	2B, PCB, PA (1 year)	(180) Postmenopausal women with T2D	27 g/day flavonoid-enriched chocolate (containing 850 mg flavan-3-ols [90 mg epicatechin] + 100 mg isoflavones [aglycone equivalents)] /d) *vs.* PCB.	Intima-media thickness of the common carotid artery, pulse wave velocity, augmentation index, BP, and vascular biomarkers	Only pulse pressure variability improved
Flavonoids (Isoflavones)	Curtis *et al.* (2013) [80]	5	PA, PCB (1 year)	(93) Postmenopausal women with T2D	27 g/day flavonoid-enriched chocolate (containing 850 mg flavan-3-ols [90 mg epicatechin] + 100 mg isoflavones [aglycone equivalents)] /d) *vs.* PCB.	HOMA and QUICKI, lipid profile, BP	Estimated 10-year total coronary heart disease risk (derived from UK Prospective Diabetes Study algorithm) was attenuated after flavonoid intervention
Flavonoids (Isoflavones)	Chan *et al.* (2008) [81]	5	2B, PCB (12 weeks)	(102) Prior ischemic stroke	80 mg/day isoflavone supplement *vs.* PCB	EF, nitro-glycerine-mediated dilatation, BP, HR, CHO metabolism, haemoglobin A1c, and oxidative stress biomarkers	↓ serum hsCRP and improved brachial EF in patients with clinically manifest atherosclerosis
Flavonoids (Isoflavones)	Webb *et al.* (2008) [82]	4	2B, PA (5 days)	(71) Subjects with CAD	Isoflavone-intact soy protein (75 mg/day of isoflavones) *vs.* isoflavone-free PCB	Stimulated coronary blood flow, Basal and stimulated coronary artery luminal diameters	No significant effect
Flavonoids (Isoflavones)	Fanti *et al. (*2006) [83]	3	2B, Crtl, prospective, pilot study (8 weeks)	(32) ESRD patients with systemic inflammation	Nutritional supplements (soy groups) containing 26–54 mg isoflavones aglycones *vs.* isoflavone-free milk-based supplements (Ctrl group)	Inflammatory biomarkers	Inverse correlation between blood isoflavones levels and CRP, positive correlation between blood isoflavones levels and IGF-1
Flavonoids (Procyanidins)	Ras *et al.* (2013) [84]	5	2B, PCB, PA (8 weeks)	(70) Healthy subjects	300 mg/day Grape Seed Extract *vs.* PCB	BP	No significant effect
Flavonoids (Procyanidins)	Yubero *et al.* (2013) [85]	3	2B, PCB, X (56 days)	(60) Healthy subjects	700 mg/day the Grape Extract (Eminol®) *vs.* PCB	CVD risk and oxidative stress markers	↓TC, LDLc and ↑ TAC and vitamin E.
Flavonoids (Procyanidins)	Asher *et al.* (2012) [86]	5	2B, PCB, four-period X (3.5 days, 4 days washout)	(21) Pre-hypertensive or mildly hypertensive adults	Hawthorn Extract (1000, 1500, and 2500 mg/day) *vs.* PCB	EF and nitric oxide release	No significant effect
Flavonoids (Procyanidins)	Liu *et al.* (2004) [87]	3	PCB, 2B, PA (12 weeks)	(58) HT subjects	100 mg/day Pycnogenol *vs.* PCB	Endothelin	↓ Calcium antagonist nifedipine. ↓ endothelin-1 concentration and ↑ of 6-keto prostaglandin F1a.
Flavonoids (Procyanidins)	Enseleit *et al.* (2012) [88]	5	2B, PCB, X (8 weeks, 2 weeks washout)	(23) Patients with stable CAD	200 mg/day Pycnogenol *vs.* PCB	EF, oxidation and inflammatory markers, platelet adhesion and 24 h BP	EF improvement. ↓ 8-iso-PGF2α
Flavonoids (Procyanidins)	Mellen *et al.* (2010) [89]	3	2B, PCB, X (4 weeks, 4 weeks washout)	(50) Patients with CAD	1300 mg/day muscadine grape seed *vs.* PCB	EF, oxidation and inflammatory markers, antioxidant status	No significant effect
Flavonoids (Procyanidins)	Tauchert *et al.* (2002) [90]	3	2B, PCB (16 weeks)	(209) Chronic stable heart failure patients	1800 mg/day crataegus extract WS 1442 or 900 mg/day crataegus extract WS 1442 *vs.* PCB	Typical heart failure symptoms	Typical heart failure symptoms as rated by the patients were ↓ to a greater extent

ªSoy extract contains: contains 17.5 mg soy isoflavones consisting of 5.25 mg glycitin, 8.75 mg daidzein, and 3.5 mg genistein; ^b^ 44 mg of daidzein, 16 mg of glycitein, and 10 mg of genistein; ^c^ Soy groups in three formats: Protein powder (54mg isoflavones), Cereal-like product (26mg isoflavones), energy bar (26 mg isoflavones). 8-iso-PGF2α, 8-iso-prostaglandin F2α; 1B, one-blind, 2B, double-blinded; ACN, anthocyanins; Apo, apolipoprotein; BMI, body mass index; BP, blood pressure; CAD, chronic artery disease; CAT, catalase; CHF, chronic heart failure; CHO, carbohydrate; CRP, C-reactive protein; hsCRP, high sensitivity c-reactive protein; Ctrl, control, CVD, cardiovascular disease; DXA, Dual-energy X-ray absorptiometry; EF, endothelial function; EGCG, epigallocatechin gallate; ESRD, European and North American end-stage renal disease; FM, fat mass; FFM, fat-free mass; HDLc, high-density lipoprotein cholesterol; HOMA, homeostasis model assessment; HR, heart rate; HT, hypertension; sICAM, soluble intercellular adhesion molecule; IGF-1, insulin-like growth factor-1; IR, insulin resistance; IL, interleukin; LDLc, low-density lipoprotein cholesterol; MDA, malonaldehyde; MPFF, micronized purified flavonoid fraction; MPI, milk protein isolate; NTG, nitro-glycerine-mediated dilation; PA, parallel design, PAI-1, plasminogen activator inhibitor-1; PCB, placebo, PBMCs, peripheral blood mononuclear cells; PS, plant sterols; PWV, pulse wave velocity; QUICKI, quantitative insulin sensitivity check index; SBP, systolic blood pressure; SOD, superoxide dismutase; TC, total cholesterol; TGF, transforming growth factor; T2D, type 2 diabetes; VCAM, soluble vascular cellular adhesion molecule; vWf, von Willebrand factor; X, crossover design. *Included after proofreading.

### 3.1. Simple Phenols

#### 3.1.1. Stilbenes

Eight articles complied with requirements and their score varied from 3–5 and are included in Table 1. The age ranged from 20 until 83 years, with sample sizes of 19–166 participants. Two studies included an acute intervention design and were carried out in overweight/obese men and post-menopausal women with elevated BP [19,20]. Participants consumed three doses of resveratrol (RSV) (30 mg, 90 mg or 270 mg) or 75 mg of trans-resveratrol [20] and one hour after supplementation, they determined possible improvement of EF. Wong *et al.* [19] observed that EF increased more with the highest dose and later confirmed the increase of flow mediated dilation (FMD) with a 75 mg dose in an acute and a long term study [20]. One study was developed in healthy smoker subjects [21], testing the efficacy of RSV (500 mg/day) on anthropometric parameters and CHO metabolism, apart from lipid profile, markers of inflammation and oxidative stress during 60 days. Thus, after RSV supplementation, hsCRP and decreased, while antioxidant status (TAS) increased. Effects of short-term oral supplementation (60 days) of RSV alone (20 mg/day) or with calcium fructoborate (CF) (20 RSV + 112 CF mg/day) in subjects with stable angina pectoris were recently evaluated by Militaru *et al.* [22], measuring lipids profile, hsCRP, left ventricular function markers and observed a stronger decreased in N-terminal prohormone of brain natriuretic peptide (NT-proBNP) after RSV+CF administration. Moreover, RSV alone presented the most significant decreases for TC and TAG, although, reduction of high sensitivity C-reactive protein (hsCRP) was greater using CF treatment (112 mg/day). Tomé-Carneiro and colleagues [23,24,25,26] reported four studies with grape extract containing RSV combination (GE-RES), comparing with grape extract alone (GE). In 2012, they developed a clinical trial in primary prevention of CVD patients. After six months with 350 mg/day GE or 350 mg/day GE-RES possible changes in lipid profile and oxidized LDL(oxLDL) were assessed [24] and then, doses were doubled for the next six months. LDLc, apolipoprotein B (ApoB), oxLDL, and oxLDL/ApoB ratio decreased in the GE-RES group, whereas non-HDLc (total atherogenic cholesterol load)/ApoB ratio increased. Moreover, they evaluated the effect in inflammatory and fibrinolytic biomarkers after one year [26]; many improvements were observed: hsCRP, tumour necrosis factor alpha (TNF-α), plasminogen activator inhibitor 1 (PAI-1), interleukin (IL) IL-6/IL-10 ratio, and soluble intercellular adhesion molecule (sICAM) significantly decreased, and IL-10 and adiponectin increased. . In 2013, they evaluated the same doses (350 mg/day GE or 350 mg/day GE-RES), for the first six months and double for the following six months in patients with T2D, HT and stable coronary artery disease (CAD) [25], as well as in patients with stable CAD alone [23]. Peripheral blood mononuclear cells (PBMCs),inflammatory andfibrinolytic biomarkers were assessed in both studies; the pro-inflammatory cytokines CCL3, IL-1β and TNF-α expression levels, were significantly reduced and transcriptional repressor LRRFIP-1 expression increased in PBMCs from T2D, HT and stable CAD patients taking the GE-RES extract [25]. In the other trial, the GE-RES group showed an increase of the anti-inflammatory serum adiponectin and PAI-1 decreased. In addition, six key inflammation-related transcription factors were predicted to be significantly activated or inhibited, with 27 extracellular-space acting genes involved in inflammation, cell migration and T-cell interaction signals presenting down regulation in PBMCs from stable CAD patients [23].

RSV has been shown to exert its protective effect against cardiovascular disease but it is necessary to reiterate that these data derive from cell culture or small animal model systems, with no reports on long-term health or survival in humans or alternate animal models [91]. It is well known that impaired FMD is recognized as an independent risk factor for the development of CVD [92,93]. Several authors investigated the acute resveratrol supplementation effect in overweight/obese individuals with mildly elevated BP, thus these subjects present cardiovascular risk [94,95]. Wong *et al.* [19] observed improvements in FMD were correlated with a dose-related increase in plasma RSV concentrations and following up their research, they confirmed the effect of RSV on FMD [20]. However, more long-term interventions are required. The other studies assessed long-term administration, until one year. Generally, trials obtained a decrease in total cholesterol (TC), triglycerides (TAG), C-reactive protein (CRP), CCL3, IL-1β, sICAM, NT-proBNP, TNF-α expression, LDLc, ApoB, LDLox and LDLox/ApoB ratio, as well as adiponectin, non-HDLc/ApoB ratio, IL-10 in different type of subjects [21,22,23,24,25,26].

Reports by Tomé-Carneiro *et al.* [23,24,25,26] focused on PBMCs, inflammatory andfibrinolytic biomarkers, lipid profile, and oxLDL concentration. Prior studies have shown increased mitochondrial production of ROS in PBMCs, endothelial cells, and other cell types in diabetes, suggesting systemic mitochondrial dysfunction [96,97]. Hartman *et al.* [98] observed higher basal, maximal, and uncoupled oxygen consumption in the diabetic patients, findings that are consistent with prior work showing increased mitochondrial ROS production in PBMCs. This suggests how serious complications may be in T2D subjects. Results by Tomé-Carneiro *et al.* [23,24] on transcriptional levels appear interesting; nevertheless, we have not found further similar interventions confirming these results.

#### 3.1.2. Catechols

Two articles about catechols were selected (Table 1). According to the Jadad scale, such studies obtained values from 4–5. In 2008, Alwi *et al.* [27] assessed effects of curcumin on lipids profile in 75 acute coronary syndrome (ACS) patients (45–73 years). The efficacy was measured using different doses (45 mg/day, 90 mg/day or 180 mg/day) during two months, reporting higher effects in TC, LDL reduction and an increase in HDL with the lower dose, but changes were not significant in respect to placebo. Recently, a study [28] also evaluated the efficacy and safety of curcumin extract (750 mg/day) as an intervention agent for reducing the risks for atherogenesis in 240 T2D patients with a mean age of 61 years, by means of parameters such as BP, anthropometry, lipids profile, adiponectin, leptin, CHO metabolism, uric acid, and pulse wave velocity (PWV). After six months, curcumin treatment significantly reduced PWV, homeostasis model assessment (HOMA), TAG, acid uric, leptin and abdominal obesity, as well as significantly elevated values of adiponectin.

Alwi *et al.* [27] developed the first study to evaluate the effect of curcumin on the lipid profile of patients with ACS), thus the antecedents are described in *in vitro* and *in vivo* animal models. Results did not change significantly when comparing with placebo group. A meta-analysis based on five clinical trials in relation to curcumin on blood lipids concentration, indicated a non-significant effect of curcumin on the lipid profile when considering heterogeneous populations including healthy subjects, obese dyslipidemic patients, elderly subjects with established acute diagnosis of Alzheimer’s disease, ACS and patients with T2D [99]. Chuengsamarn *et al.* [28] also assessed lipid profiles and did not find significant statistic differences compared with placebo. Ramirez-Boscá *et al.* [100] observed that a daily treatment with curcumin extract could decrease significantly the LDLc and ApoB concentrations and increase the HDL and ApoAI in healthy subjects. However, due to lack of sufficient data we cannot recommend curcuminoids for improvement lipids profile in healthy and unhealthy subjects until further solid evidence is obtained.

On the other hand, after six months of curcumin intervention [28], PWV, HOMA, TAG, uric acid, abdominal obesity and leptin decreased, in addition to adiponectin increase. In addition, curcumin was well tolerated, with very few adverse effects. Agreeing with that, curcumin administration has been demonstrated, in *in vitro* and *in vivo* animal models, to elevate adiponectin and to decrease leptin levels [101,102], and oxidative stress in rabbits [14].

Due to its benefits and safety, Chuengsamarn *et al.* [28] proposed that curcumin extract might be used as anti-atherosclerotic in T2D populations. We propose however to replicate these results in other populations, since this study was performed in a Thai population and high variabilities of physical activity and diet among populations may exist that affect study results. Moreover, there are not enough studies in humans for recommending curcumin against T2D.

#### 3.1.3. Beer or Wine Polyphenols

Four of the studies were focused on the effects of polyphenols derived from beer or wine; all were in subjects with risk of CVD (55–75 years) (Table 1). They tested 280 mg of red wine polyphenols or 30 g/day of beer or wine (normal and dealcoholized) for four weeks [29,30,31,32]. Botden *et al.* [29] analysed BP and found no significant effect. Chiva-Blanch *et al.* [30,31,32] reported that after the beer and non-alcoholic beer interventions the number of circulating endothelial progenitor cells (EPC)-mobilizing factors increased, consumption of dealcoholized red wine decreased BP and alcohol increased IL-10 and decreased macrophage-derived chemokine concentration and that the phenolic compounds of red wine decreased the serum concentrations of ICAM-1, E-selectin and IL-6.

Botden *et al.* [29] studied the effects of polyphenols from wine on BP and found no effect except that, the use of dealcoholized red wine reduced BP. Moreover, phenolic compounds are related to decreases of inflammatory and vascular homeostasis biomarkers. Nevertheless, there is not strong evidence showing that consumption of beer or wine could help to improve risk of CVD; reports in the literature do not focus on a specific compound, regardless of alcohol content. A review by Rotondo *et al.* [103] states that wine in low quantities could be beneficial in regard to CVD, but notes possible bias in the publications reviewed. It is necessary to focus research on a specific compound in alcohol-containing products, when assessing for potential benefits in CVD.

### 3.2. Polyphenols

The different research equations resulted in 59 articles related to different flavonoids and subclasses, such as anthocyanins (ACN), flavonols, flavanols, isoflavones and procyanidins. Eight of the studies were discarded because of lack of information in the abstract or inability to obtain the full-text version. The results are presented in groups according to their class in Table 2 and Table 3.

#### 3.2.1. Anthocyanins

Five publications were related to ACN (Table 2). Quality scores for these studies ranged from 4 to 5 in the Jadad scale. Two of the studies were exclusively in women between 23 and 58 years [33,34] (sample sizes from 31 to 57) and one, in 31 hypertensive men aged between 35 and 51 years [35]. The study developed by Dohadwala and colleagues [36] was in patients with CAD using an acute and a chronic approach.

The doses of ACN provided were from 500 to 640 mg/day [97,98] alone or as juice or blended drink including 94 mg/day [36], 983.7 mg/day and 840.9 mg/day [33], respectively, during periods from 14 days to 4 weeks.

The main outcomes in these studies were related to BP, lipid profile, CHO metabolism, inflammatory and oxidative stress biomarkers, platelet reactivity and vascular function.

One of the studies reported an increase of HDLc and blood glucose after the ACN intake, but no effects on oxidative stress biomarkers [35]. On the other hand, Kuntz *et al.* [33] reported an increase on superoxide dismutase (SOD) and catalase (CAT) and a decrease of malonaldehyde (MDA) after the ACN ingestion. The other studies did not find any significant effect when analysing BP, CHO metabolism, lipid profile, inflammatory biomarkers, platelet reactivity [34] or vascular function [36].

A recent systematic review has shown the effectiveness of anthocyanins in decreasing CVD risk [104]. Nevertheless, in this review we found that improvement of risk factors related to CVD such as BP, lipids profile, CHO metabolism, inflammatory, oxidative stress biomarkers, and platelet reactivity were not consistent. Hassellund *et al.* [35] reported modifications in lipid and CHO metabolism, but this result was not supported in the other investigations, as was the case with oxidative stress as well. There is not strong evidence supporting that anthocyanins help to decrease risk of CVD and further studies are required, thus, the grade of recommendation according to the SIGN guidelines is B.

#### 3.2.2. Catechins

Four of the studies were related to catechins (Table 2), and the quality score assigned according to the Jadad scale was around 3–5. Two of the studies were with healthy subjects between 32 and 69 years old [37,38] (sample size 52 and 64 in each one). In regard to the other two studies, one included 52 subjects with early atherosclerosis (mean of 42 years of age) [39], and the other included 240 subjects with visceral fat-type obesity aged around 25–55 years old [40].

In the studies with healthy subjects [37,38] and the one in visceral fat-type obesity [40], catechins were obtained from green and black tea, with doses between 583 mg and 3 g per day during periods between 4 and 14 weeks. The atherosclerotic subjects [39] were supplemented with 30 mL of epigallocatechin gallate (EGCG)-supplemented olive oil during 4 months.

The main outcomes in the healthy subjects [37,38] were related to CVD risk, such as inflammatory and endothelial biomarkers. Subjects with early atherosclerosis [39] were investigated in regards to endothelial function and inflammatory and oxidative stress status. The aims in the subjects with visceral fat-type obesity [40] were anthropometric measurements, body fat composition and CVD risk factors.

There were no significant effects of the use of catechins in healthy subjects when compared with placebo/control; however, there was a negative correlation between beta-carotene and the inflammation biomarkers, IL-6 and fibrinogen [38]. On the other hand, the intervention in visceral fat-type obesity showed significant decreases in body weight, body mass index (BMI), body fat ratio, body fat mass, waist circumference (WC), hip circumference, visceral fat area and subcutaneous fat area, systolic blood pressure (SBP) and LDL cholesterol [40]. Nevertheless, in patients with early atherosclerosis, there was no significant effect, but merging both, the control (olive oil) and the experimental group (olive oil and EGCG)) the endothelial function was improved [39].

Catechins were shown to be effective in reducing LDLc and TC, but there is no robust evidence in reducing CAD risk [105]; a dose of 583 mg of catechins in middle-aged subjects showed a significant effect reducing obesity related makers, such as body weight, BMI, body fat ratio, body fat mass, WC, hip circumference, visceral fat area, and subcutaneous fat area, SBP, LDLc. However, doses of 630 mg or 3 g did not benefit middle and older aged subjects. Furthermore, Widmer *et al.* [39] investigated the effects of olive oil with EGCG in endothelial function without significant effect; however, they found a significant improvement when they merged both study groups. Nevertheless, this result is attributable to olive oil compounds, independently of the EGCG content. Taking into account the different studies included in this review, we can conclude that there is no robust evidence to suggest a beneficial effect of tea catechins on prevention of CVD; consequently, the grade of recommendation according to the SIGN guidelines is B.

#### 3.2.3. Flavanols

Fourteen investigations studied the effects of flavanols (Table 2); the Jadad quality scores were between 3 and 5. Only one study was in healthy males (mean 68 years, and with a sample size of 40) [41], while four papers were related to overweight adults (with one of them including two designs and thus treated as separated studies [45]), involving subjects from 40 to 64 years (*n* = 21–98) [42,43]. Furthermore, there were two studies in hypertensive subjects [46,47] with 52 and 19 patients respectively. The study developed by Flammer *et al.* [48] included 20 chronic heart failure (CHF) patients (58 years mean) with an acute and a long-term intervention. Heiss *et al.* [49] and Horn *et al.* [50] studied the effect of flavanol in 16 CAD patients (60 years mean) while Balzer *et al.* [51] designed a study in diabetic patients with an acute and long-term intervention (10–41 subjects between 50 and 80 years).

Four of the studies [42,45,48,51] used a short-term approach looking for the acute response of flavanols; they tested cocoa or chocolate in amounts from 624 mg to 963 mg/day. Further, the other studies tested the flavanols contained in chocolate in longer interventions, from 4 to 6 weeks in doses from 33 to 1052 mg/day [43,44,46].

The aim of the study in healthy males [41] was to determine the effect on the endothelial function and in the soluble cellular adhesion molecules, without significant effects. In regards the studies with overweight [42,43,45], hypertensive [46,47], CHF [48], CAD [49,50] and diabetic patients [51], the main outcomes were related to EF and BP. Furthermore, Grassi *et al.* [47] studied the effects on lipids profile and IR.

The results in overweight, HT, CHF, CAD and diabetic patients showed a consistent improvement in EF when comparing different doses of flavanols *vs.* placebo/control [42,43,44,45,47,48,49,50,51].

When testing flavanols in BP, Faridi *et al.* [29] found a significant decrease in BP in overweight adults in an acute intervention with two different products using >800 mg/day of flavanols. Additionally, BP decreased significantly in hypertensive subjects using flavanol-rich chocolate during 15 days [47] and in CAD patients [49] with 375 mg twice daily during 30 days. However, this result was not consistent when using doses between 33 and 1052 mg/day of flavanones during six weeks [46] or 814 mg/day during four weeks [44]. Besides, Berry *et al.* [42] found out that cocoa flavanols could attenuate the increase of BP after exercise. The effects on IR were investigated in two studies, finding a significant improvement [43,47].

Flavanol-rich chocolate and cocoa products have shown a small but statistically significant effect in lowering blood pressure by 2–3 mm Hg in the short term [101], in addition Khawaja *et al.* [106] suggest that there is ample evidence in support of the beneficial effects of cocoa/dark chocolate on CHD risk. Summary of the evidence showed benefits of cocoa flavanols in BP and EF, in overweight adults [42,43,44,45,46], hypertensive subjects [47], in CHF [48], in CAD [49,50] and in T2D patients [51] utilizing different doses (149–963 mg/day). Additionally, Balzer *et al.* [51] reported a dose-response in an acute intervention. While the use of cocoa flavanols in BP and EF improvement have shown efficacy, further studies using flavanol-free controls could help to strength the evidence and long-term interventions may clarify the effect on CVD, thus the grade of recommendation according to the SIGN guidelines is B.

#### 3.2.4. Flavonols

Five studies were focused on the effects of flavonols on CVD (Table 2); all of them obtained more than 3 points in the Jadad scale. Three of the publications were focused on healthy males [52,53,54] aged between 24 and 53 years (sample size between 12–27). The other two studies studied the effect on hypertensive subjects [52,55] (24–49 years; including 12 and 41 subjects, each one). Quercetin was the flavonol tested in two of the healthy subject studies at a dose of 1 g/day; Larson *et al.* [52] used it in an acute study and Conquer *et al.* [53] used it for 28 days; both of the authors looked for effects in BP and vascular markers without effect. Moreover, Suomela *et al.* [54] utilized oatmeal with 78 mg of flavonol aglycones from sea buckthorn for four weeks, and likewise did not find any significant effect.

The studies focused on hypertensive subjects looked for effects in BP, oxidative stress, angiotensin-converting enzyme and endothelin. Both, Edwards and Larson [52,55] found a significant reduction in BP in hypertension. Evidence around flavonol has been controversial; previous meta-analyses has associated its consumption with lower rates of CHD [8] or a reduction in risk of stroke [107,108], but reports from other authors do not support the protective role against CHD [10]. In the present review, we have found that doses of 1 g/day of quercetin or an oatmeal with 78 mg of aglycones of quercetin did not shown effects on diverse CVD risk markers, such as endothelin, BP or oxidative stress. Nevertheless, an acute intervention with 1095 mg/of quercetin and a long-term intervention (28 days) with 730 mg/day seems to be effective at reducing BP in hypertensive men [52,55], but not on other oxidative stress or endothelial function markers. We can conclude that there is no effect of flavonols in CHD, thus the grade of recommendation according to the SIGN guidelines is B, but since it seems that flavonols are effective at reducing BP in hypertensive men, further analysis in greater cohorts are needed.

#### 3.2.5. Isoflavones

Thirty papers were related to the consumption of isoflavones (Table 3), the Jadad scores were above three points. Five of the studies were in healthy subjects between 20 and 53 years (sample size 22–205) [56,57,59,60,68]. Most of the studies were developed in postmenopausal women (45–92 years) with normal weight [61,62,63,64,65,66,67], overweight and obese [69,70,71], with BP alterations [78,109], dyslipidaemias [76,77,79] or T2D [80], including from 40 to 350 participants. Other authors also took men in account [58,72,73,74,75]. One study investigated in 102 subjects prior to ischemic stroke [81] (mean 66 years), another in 71 subjects with CAD (mean 58) [82] and Fanti and colleagues studied the effect on patients with systemic inflammation [83].

The doses of isoflavones employed in healthy subjects and postmenopausal women were between 40 and 118 mg/day, with lengths between 17 days to two years. The intervention in postmenopausal women with T2D used 100 mg/day of isoflavones for a one-year period. The subjects with ischemic stroke consumed 80 mg/day of isoflavones for 12 weeks. Patients with CAD included 75 mg/day during five days. Subjects with systemic inflammation included doses between 26–54 mg/day of isoflavones aglycones [56,57,58,59,60,61,62,63,64,65,66,67,68,69,70,71,72,73,74,75,76,77,78,79,80,109].

The main outcomes established in the articles of healthy, overweight and obese subjects were related to anthropometry [66], body composition [63,70,71,109], lipid profile [56,57,58,59,60,62,63,65,66,67,68,69,70], BP, CHO metabolism [60,62,63,66,70], and inflammatory [57,59,63,64,67], oxidative stress [62] and vascular homeostasis biomarkers [59,62,64] and only one in atherosclerosis progression [61]. Besides, the studies in hypertensive and dyslipidemic patients aimed on lipids profile [73,74,76,79], BP [73,75,76,78,79], oxidative stress [72,74,76], endothelial function [75,78] and just one in vascular inflammation biomarkers [77]. Two articles from Curtis *et al.* [80,110] in postmenopausal women with T2D looked for effects in HOMA, QUICKI, lipids profile, intima-media thickness of the common carotid artery, pulse wave velocity, augmentation index, BP, and vascular biomarkers.

The results reported in healthy, overweight and obese patients related to anthropometry and body composition were without significant effects of isoflavones. Moreover, in relation with lipids profile, a study reported lower ratios of TC/HDLc, LDLc/HDLc, and ApoB/Apo A-I [56] and another a decrease in TC and LDLc [71]. Besides, Sanders *et al.* [57] found significant improvements in HDLc and Apo-A; however, seven studies did not find any significant effect in plasma/serum lipids [58,59,60,63,65,66,67,69]. Fasting glucose, insulin, and HOMA were reduced in three studies [59,60,66] but results from other authors were not consistent [63,69,70]. The only author that studied BP [63] did not find any significant change. Atteritano *et al.* [62] reported significant improvements in isoprostanes (8-iso-PGF2α), sICAM-1 and soluble vascular cell adhesion molecule-1, while Atkinson *et al.* [59] and Sanders [57] investigated PAI-1 and other authors [72,74] 8-iso-PGF2α without significant results. One study showed a decrease in thromboxane A2 [63] and another in CRP [64]. The study of Liu *et al.* [67] aimed in inflammatory markers showed no effect. Hodis *et al.* [61] analysed atherosclerosis progression finding no positive effects. Furthermore, in subjects with BP alterations and dyslipidaemias, there were no significant changes in CHO metabolism [79,80], oxidative stress or inflammatory biomarkers [72,74,76,77]. In relation to BP, Sagara *et al.* [73] and Teede *et al.* [78] reported improvements; nevertheless, these results were not consistent with the results obtained by other authors [75,76,79,80]. Moreover, when lipid profile were analysed, a decrease in total cholesterol, LDLc and a decrease was observed [73,76,79] but Meyer *et al.* [75] and Thorp *et al.* [58] reported no significant change. Many authors [52,56,57,108] described no significant effects on endothelial function; nonetheless, Jenkins *et al.* [76] reported a lower calculated risk of CAD. The intervention in subjects’ prior ischemic stroke [81] found a reduction in hsCRP and improved the EF, beside CAD patients [82] showed no effect on EF. Fanti *et al.* [83] found a significant inverse correlation between isoflavones and CRP.

The use of isoflavones on the prevention of CVD has been associated with the capacity of these compounds to attenuate alterations in lipid profile and inflammatory markers [111,112]. Furthermore, the American Diet Association recommended consumption of soy protein containing isoflavones in high-risk populations with increased total cholesterol and LDLc. Additionally, data from two cohorts [113,114] showed that isoflavones consumption is associated with a lower risk of cardiovascular disease in women. Interestingly, we found that doses above 50 mg/day in healthy subjects improved lipids profiles [56,57,68], but a lower dose for 12 months did not show effect on CVD risk factors [45]. Additionally, normal weight postmenopausal women did not improve the lipid profile in many of the studies in which they included as outcome [60,63,65,66,67,69]. Nevertheless, the response of dyslipidemic postmenopausal women was positive when treated with doses between 60 and 80 mg/day of isoflavones, decreasing TC, HDLc and ratios TC/HDLc, LDLc/HDLc and ApoB/A-I [76,79].

Glucose, insulin and HOMA measurement are important due to its relationship in development of CVD. Although, these indicators were reduced in normal weight subjects [41,43,49], isoflavones in high doses (60–100 mg/day) showed no benefit on overweight/obese postmenopausal women [63,69,70,79].

BP was only measured by Garrido *et al.* [63] in healthy subjects without significant changes. A different panorama was shown in subjects with BP and dyslipidaemias, while doses above 80 mg improved BP [73,78], doses below did not have any effect [75,76,79,80].

The research focused on inflammatory and oxidative stress biomarkers in healthy subjects gave inconclusive results, markers such as fibrinogen and PAI-1 were not reduced, but only two authors measured them [57,59]. Thromboxane A2, CRP, and 8-iso-PGF2α were markers that also responded effectively to isoflavones intervention, but were not taken in account in all the investigations [57,59,60,62,63,64,67,71]. Moreover, Liu *et al.* [109] focused his research in measuring inflammatory markers after an intervention with soy or milk protein plus 100 mg of isoflavones without significant effects. This lack of consistent results was already showed by Dong and colleagues [114] in a meta-analysis including 14 trials that analysed soy foods with isoflavones concluded that there is insufficient evidence that soy isoflavones significantly reduce CRP concentrations in postmenopausal women.

Dysfunction of the vascular endothelium has shown to be an early step prior to development of atherosclerosis [115], its prevention is vital for the maintenance of vascular health. Some authors reported no EF improvement [65,74,75,80], indeed, Webb *et al.* [82] conclude a lack of effect on EF in their intervention; however, the inclusion of more women to perform a specific gender analysis could give different results. Nevertheless, Chang *et al.* demonstrated that 12-week isoflavone treatment improved brachial FMD in patients with clinically manifest atherosclerosis, thus reversing their endothelial dysfunction status. In this regard, Li *et al.* [9] developed a meta-analysis measuring isoflavones on vascular endothelial function in postmenopausal women and conclude that oral isoflavone supplementation does not improve endothelial function in postmenopausal women with high baseline FMD levels but leads to significant improvement in women with low baseline FMD levels. Furthermore, Pase *et al.* [116] determined that soy isoflavone supplementation provides an effective means of reducing arterial stiffness, but this review showed to be biased.

Lastly, the use of isoflavones in body composition and anthropometric measurements showed no effect in most of the interventions that included study of outcomes [63,66,67,69,70]. In summary, the need of further studies with greater population sizes are necessary; the effect of the inflammatory process and endothelial function in postmenopausal women on are possibly the most interesting areas of study. The grade of recommendation according to the SIGN guidelines is B.

#### 3.2.6. Procyanidins

Eight studies from the search were related to procyanidins (Table 3), with a Jadad score above three points. Two interventions were in healthy subjects between 34 and 75 years [84,85], sample size 60 and 70, in each one. Asher *et al.* [86] and Liu [87] studied the effects of procyanidins in HT and the three other studies were in CAD (18–73 years, sample size of 23 and 50) and the last study included 209 chronic stable New York Heart Association class-III heart failure subjects [90].

The interventions in healthy subjects used 300–700 mg/day of grape extracts with a length of eight weeks in both [84,85]. In HT subjects, the hawthorn extract was investigated in a short tem study of three and a half days in doses of 1 g, 1.5 and 2.5 g [86]. In the other study they used 100 mg/day of Pycnogenol^®^ during 12 weeks [87]; this product was also utilized at a dose of 200 mg/day in stable CAD patients for eight weeks [88]. Besides, muscadine grape seed was used for four weeks in doses of 1300 mg/day [89]. The subjects with stable CHF were treated with 1800 mg of crataegus extract WS 1442 or 900 mg of crataegus extract WS 1442 or with placebo for 16 weeks [90].

In healthy subjects, the outcomes were related to BP, CVD and oxidative stress biomarkers, in hypertensive subjects to endothelial function, in CAD to endothelial function, inflammatory and oxidative stress biomarkers. In patients with stable CHF, the aim was related to typical heart failure symptoms [84,85,86,87,88,90].

There were no significant effects in BP in healthy subjects [84]. However, Yubero *et al.* [85] reported a significant decrease in TC, LDL and an increase in TAC and vitamin E. Furthermore, in hypertensive subjects, Asher *et al.* [86] did not find significant effects, while on the contrary Liu *et al* [87] found a reduction of endothelin-1 concentration and an increase in 6-keto prostaglandin F1a. Moreover, the use of Pycnogenol in CAD also showed an improvement of endothelial function and a reduction of 8-iso-PGF2α [88]. The muscadine grape seed did not show any significant effect [89] and the crataegus extract seemed to reduce the typical heart failure symptoms [90].

Procyanidins are compounds that can stabilize membranes, preventing their disruption by chemical and biological agents, thus mitigating oxidative stress and the activation of proinflammatory signals, factors related to development of CVD. Evidence found in the literature reviewed is not consistent. In healthy subjects, there was no effect on BP [84] using 300 mg/day of grape seed extract. However, Yubero *et al.* [85] reported improvement on CVD risk and oxidative stress markers with 700 mg/day. Besides, in hypertensive subjects, one study including different doses showed no improvements in EF or NO release, while another with 100 mg of Pycnogenol^®^ reduced the concentration of endothelin and 6-keto prostaglandin F1a. The same compound was utilized in a dose of 200 mg/day in patients with stable CAD with improvements in EF and a decrease in isoprostanes. However, Mellen *et al.* [89] studied the effect of muscadine grape seed (1300 mg) without further effects. Finally, Tauchert *et al.* [90] included two different extracts from crataegus in typical heart failure symptoms with a positive decrease as rated by patients. In this regard, there is insufficient evidence to determine if extracts containing procyanidins could improve CVD risk; further investigations are necessary for more homogenous outcomes and greater populations; therefore, the grade of recommendation is C.

## 4. Limitations and Future Perspectives

Certain limitations need to be considered. Firstly, MeSH terms are not often used by researchers. Such specific terms must be taken into account when articles are drafted and assuring a good indexation and more visibility, facilitating the evidence valuation. Secondly, the application of resources such as CONSORT (Consolidated Standards of Reporting Trials) statement or Jadad scale is highly scarce. Moreover, the existence of checklists helps authors and editors to improve the reporting of RCTs and consequently provides scientific quality in data reports. We consider that a clinical trial is reliable when it is at least randomized and blinded. In addition, the trials included in this review have high levels of heterogeneity, making it more difficult to draw concrete conclusions in relation to types of subjects, form of analysed product or its combination with other compounds.

Future studies must show better designs to avoid the risk of bias usually associated with potential confounding variables such as other dietary or lifestyle factors. In addition, the dosages, polyphenol type, duration and frequency of consumption must be clear, giving the opportunity to assess the possible benefits to a specific compound. Long-term, double-blind, crossover, randomized clinical trials with specific clinical endpoints should be developed to guarantee the possible benefits of phenolic BAC.

In addition, the use of potent new technologies such as omics sciences *i.e.* transcriptomics, metabolomics, could help to elucidate the different mechanisms in which BAC are involved in CVD and its specific role.

## 5. Conclusions

The role of BAC as adjuvants in CVD is increasing and validation of its effects is essential. Evidence shows that some polyphenols used as BAC such as flavonols are helpful in decreasing risk factors of CVD. However, it is necessary to develop better quality RCTs (crossover design, double-blinded, long term, placebo/controlled) as well as elaborate rigorous meta-analysis of existing evidence to support the effect of BAC on the prevention and treatment of CVD.
